# Use of electronic nicotine delivery systems by pregnant women II: Hair biomarkers for exposures to nicotine and tobacco-specific nitrosamines

**DOI:** 10.18332/tid/105387

**Published:** 2019-06-06

**Authors:** Melissa M. Clemens, Victor M. Cardenas, Lori A. Fischbach, Ruiqi Cen, Eric R. Siegel, Hari Eswaran, Uwemedimbuk S. Ekanem, Anuradha Policherla, Heather L. Moody, Everett F. Magann, Gunnar Boysen

**Affiliations:** 1 Department of Environmental and Occupational Health, Winthrop P. Rockefeller Cancer Institute, University of Arkansas for Medical Sciences, Little Rock, United States; 2 Department of Epidemiology, Fay W. Boozman College of Public Health, University of Arkansas for Medical Sciences, Little Rock, United States; 3 Department of Biostatistics, Fay W. Boozman College of Public Health, University of Arkansas for Medical Sciences, Little Rock, United States; 4 Department of Obstetrics and Gynecology, College of Medicine, University of Arkansas for Medical Sciences, Little Rock, United States; 5 Department of Community Health, University of Uyo, Uyo, Nigeria

**Keywords:** pregnancy, biomarkers, hair, electronic nicotine delivery systems, smallness for gestational age

## Abstract

**INTRODUCTION:**

Public awareness of electronic nicotine delivery systems (ENDS) has increased over time, and the perception that ENDS offer a safer alternative to cigarettes may lead some pregnant women to use them to reduce cigarette smoking during pregnancy. No previous studies have used metabolite levels in hair to measure nicotine exposure for ENDS users during pregnancy. We aimed to measure and compare levels of nicotine, cotinine, and tobacco-specific nitrosamines (TSNAs) in hair samples from pregnant women who were current ENDS users, current smokers, and current non-smokers. We also aimed to estimate the association between ENDS use/smoking and smallness for gestational age (SGA).

**METHODS:**

We used hair specimens from pregnant women who were dual users (ENDS and cigarettes), smokers, and non-smokers from a prospective cohort study to estimate exposure to nicotine, cotinine, and TSNAs. The exposure biomarkers and self-reports of smoking and ENDS use were used in log-binomial regression models to estimate risk ratios (RRs) for SGA among offspring.

**RESULTS:**

Nicotine concentrations for pregnant dual users were not significantly different from those for smokers (11.0 and 10.6 ng/mg hair, respectively; p=0.58). Similarly, levels of cotinine, and TSNAs for pregnant dual users were not lower than those for smokers. The RR for SGA was similar for dual users and smokers relative to nonsmokers, (RR=3.5, 95% CI: 0.8–14.8) and (RR=3.3, 95% CI: 0.9–11.6), respectively. Using self-reports confirmed by hair nicotine, the RR values for dual ENDS users and smokers were 8.3 (95% CI: 1.0–69.1) and 7.3 (95% CI:1.0–59.0), respectively.

**CONCLUSIONS:**

We did not observe lower levels of nicotine, cotinine, and TSNAs for current dual users compared to smokers during pregnancy. The risk of SGA for offspring of pregnant dual users was similar to that for offspring of pregnant smokers. Future studies are needed to further estimate the magnitude of the association between ENDS use and smallness for gestational age.

## INTRODUCTION

The use of products containing nicotine are changing due to the development and marketing of electronic nicotine delivery systems (ENDS)^1^. ENDS are battery-powered devices that heat e-liquids (typically containing nicotine) to create aerosols; some examples of ENDS are e-cigarettes, personal vaporizers, and e-cigars^2^. Tobacco companies market ENDS as cessation aids or ‘safer’ alternatives to cigarettes, but the health consequences of ENDS use have yet to be elucidated and the contents of these devices are not fully regulated. Additionally, women are using ENDS while pregnant, and most (75%) of these women are dual users (i.e. they use ENDS and smoke cigarettes)^3,4^. According to one study, most ENDS users believe that the devices are less harmful than analog cigarettes to both mother and baby^5^, and some may substitute ENDS use for cigarette use during pregnancy.

Levels of toxicants in e-liquids may not actually translate to human exposure to nicotine and tobacco-specific nitrosamines (TSNAs) from ENDS. Public health advocates are in a difficult position when assessing the risks of these devices due to the lack of standardization and regulation^6-8^. With the addition of flavoring agents, ENDS vapor becomes a complex mixture of toxicants. Different ENDS may contain different levels and frequencies of impurities, including TSNAs, solanesol, volatile organic compounds, polycyclic aromatic hydrocarbons, phenolic compounds, and carbonyl compounds^9^. For example, TSNAs such as nicotine-derived nitrosamine ketone [4-(methylnitrosamino)-1-(3-pyridyl)-1-butanone; NNK] and its metabolite [4-(methylnitrosamino)-1-(3-pyridyl)-1-butanol; NNAL] are known carcinogens that have been detected^10-13^ in the aerosols, e-liquids, and cartridges of ENDS at levels ranging from trace amounts to 28.3 μg/L. Although the research has been limited, the data indicate a significant decrease in exposure to TSNAs for ENDS users compared to cigarette smokers in non-pregnant populations^14,15^.

A recent review of the literature found no validated biomarker specific for ENDS use^16^. Included among the most common biomarkers developed for cigarette smoking are levels (quantitated with mass spectrometry) of nicotine, cotinine, NNK, and NNAL in urine, serum, saliva, and hair^16^; each measure has advantages and disadvantages. The short half-lifes of cotinine and NNAL limit their application to assessment of only the most recent and current exposures. Furthermore, metabolism and smoking behaviors change dramatically during pregnancy, so comprehensively measuring these biomarkers during fetal development would require multiple blood, saliva, or urine samples obtained over the time-period of interest and/or repeated collection of 24-hour urine specimens. These sampling schedules and techniques would place additional burden on pregnant participants, which would complicate the study and reduce its feasibility. In contrast, nicotine concentrations measured in hair represent cumulative exposure. Nicotine concentrations in hair have been shown to correlate with both active and passive cigarette smoking exposure; levels >2.77 ng nicotine/mg hair indicate an active smoker^17,18^. Biological samples increase the likelihood of accurately measuring nicotine exposure, especially in populations in which smoking is underreported, such as among pregnant women^6,19^. There is a lack of studies that have measured nicotine, cotinine, NNK, and NNAL in hair samples of pregnant women who used ENDS.

Cigarette smoking during pregnancy increases the risk of adverse birth outcomes such smallness for gestational age (SGA)^20^. Although ENDS contain fewer chemicals, the toxicity from ENDS-derived nicotine remains a major concern because it could lead to currently unknown adverse effects that may affect ENDS users, bystanders, and, if used during pregnancy, developing infants^9,21^. In animal studies, nicotine alone can disrupt early brain development—specifically in the hippocampus—which may not be evident until adolescence^22-24^. However, due to the seemingly endless and ever-changing types, designs, and formularies, exposures to ENDS-related harmful chemicals are expected to vary widely^25^. Data on the long-term effects of TSNAs in ENDS are extremely limited, and, to our knowledge, none has examined the effects of dual use (i.e. ENDS use and cigarette smoking) by nicotine levels on birth outcomes such as smallness for gestational age.

The objectives of this study were to compare mass-spectrometry-based measures of nicotine, cotinine, NNK, and NNAL in hair samples of pregnant women according to self-report use of ENDS and cigarette smoking. Further, we aimed to examine potential differences in the relative risk for having an offspring with SGA by ENDS/smoking status as determined by validated cutoff values of hair nicotine.

## METHODS

### Design

We collected hair samples from a subset of subjects recruited for a larger study assessing the frequency with which pregnant women use ENDS, described in detail elsewhere^26^. Subjects were pregnant women who self-reported as current ENDS-only users, current smokers, current dual users (i.e. users of cigarette and ENDS) or current non-smokers. We analyzed the hair samples for biomarkers of exposure to ENDS and cigarettes (i.e. nicotine, cotinine, and TSNAs), and compared levels of the markers among the groups of pregnant women. Using a prospective cohort design, we then examined the risk for smallness for gestational age for the offspring of the women according to nicotine levels measured in hair samples and self-reported data.

### Setting and participant selection criteria

**Figure 1:**
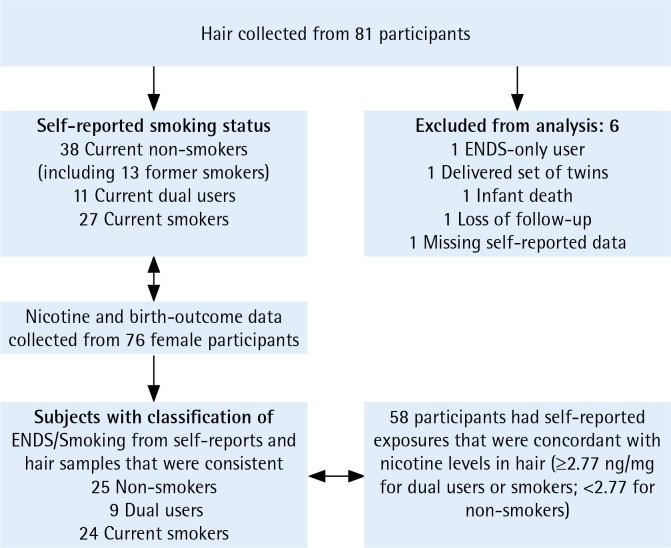
Participant inclusion and exclusion criteria

We collected samples from women who were provided care by the Women’s Clinic at a university-affiliated medical center in Little Rock, Arkansas. We included pregnant women ≥18 years of age, and we excluded women from whom we did not collect hair specimens or who gave birth to twins or other multiples. Briefly, 248 pregnant women were recruited in 2015–2016, as described in figure 1 of our companion article^26^, with 81 participants providing hair samples. We collected complete outcome data, including birth weights and gestational ages at delivery, for 76 singleton live births from these 81 participants. The group of current ENDS-only users comprised only one participant, so this group was excluded from the birth-outcome analysis because of small sample size, resulting in 76 women (instead of 77) included in our current analytical sample. All exclusions for the current analysis are described in Figure 1, where all exclusions for the current analysis are also described. All offspring births for these 76 women with hair samples occurred in 2016, while women without hair samples in the larger study^26^ were recruited in the last 6 months of 2016 and into early 2017 with completed follow-up in 2017.

### Collection of self-report data and hair samples

This study was approved by the Institutional Review Board (Protocol Number 203805) of the authors’ University. Pregnant women completed a short questionnaire to determine eligibility and gave informed consent. At a subsequent visit during the end of the first trimester or in the early second trimester of each participant’s pregnancy (mean ± SD = 18 ± 0.61 weeks of gestation), we interviewed the participant and collected a hair sample (approximately 1 inch of hair was cut nearest the scalp). As hair grows at approximately 1 cm in length per month, one cm from the scalp represents the exposure from approximately the past month, and hair samples are easier to obtain and handle than biological fluids^27-30^. The questions inquiring about ENDS were: 1) ‘Have you ever “vaped” using e-cigarettes, e-cigs, electronic cigarettes, e-hookahs, mods, pods, cartomizers, clearomizers, or tanks?’, and 2) ‘During the past 30 days, on how many days did you use an electronic vapor product?’. Participants were also provided with visual aids depicting the devices^26^. Study groups were as follows: current dual use (ENDS and cigarette smoking), current cigarette smoking, and current non-smoking. Current use was defined as reported use within the past month.

### Outcome measures

For each infant born to a mother enrolled in the study, we used their birth weight and estimated gestational age at delivery to identify smallness for gestational age (SGA). Infants who fell below the 10th percentile of the US gestational age and fetal sex-specific birth weight distribution were classified as having SGA^31^. Therefore, SGA adjusts for gestational age at delivery and fetal sex.

### Biomarker analysis

Nicotine, cotinine, NNK, and NNAL, in hair samples was quantitated as described previously^32-36^. Because hair length grows at a rate of 1 cm per month, biomarker measurements in 3 cm of hair (taken close to the scalp) represent cumulative exposures from a single trimester, which is suitable for the purposes of the present study. Each sample was analyzed with HPLC (Agilent G6490A QQQ Series) tandem mass spectrometry^33-36^.

Hair nicotine, cotinine, NNAL, and NNK, were evaluated for recovery, reproducibility, and precision on the basis of per cent recovery, coefficient of variance, and standard deviation, respectively. The validation included four sets of hair samples (three samples per set) in the following groups: positive control, negative control, smoker control, and non-smoker control. For the positive control group, 20 mg of hair from non-smokers was spiked with 50 μL of internal standard (40 pg/μL) and compounds of interest at three different concentrations (0, 12.3, 37.0 and 111 pg/μL). For the negative control group, 20 mg of hair from non-smokers was spiked with 50 μL of internal standard. Samples from known smokers and non-smokers comprised smoker and non-smoker control groups and were spiked with 50 μL of internal standard. In addition to internal validation, background signals were evaluated by running methanol blanks before each batch of specimens. On the basis of previous studies, we used ≥2.77 ng nicotine per mg hair as a cutoff point to indicate an active smoker^20-21^.

### Data analysis

Nicotine and cotinine concentrations in hair samples were transformed to their natural logarithms for statistical analysis. NNAL and NNK concentrations in hair samples were analyzed as follows. Non-detects were coded as zero for below the limit of detection (i.e. <LOD). For reporting geometric means, medians, quartiles, and ranges, <LOD was an admissible value. The presence of differences in geometric means and medians were tested using Student t-tests.

Because SGA is a not a rare outcome, we estimated risk ratios (RR) for SGA by using log-binomial regression analyses instead of logistic regression. SGA, by definition, adjusts for the offspring’s gender and gestational age at delivery. We also considered maternal age, education, and race/ethnicity as potential confounders if they changed the resulting RR by ≥10%. Concentrations of biomarkers in hair samples were used to confirm that non-smokers were not exposed to nicotine, cotinine, and their respective metabolites, in the last 3 months (the hair samples reveal cumulative exposures over approximately 3 months).

Risk ratios for SGA were obtained for the following comparison groups: 1) self-reported current ENDS users versus self-reported current non-smokers, 2) self-reported current cigarette smokers versus self-reported current non-smokers, 3) nicotine levels ≥2.77 ng/mg versus <2.77 ng/mg, 4) self-reported current ENDS users with nicotine levels ≥2.77 ng/mg versus self-reported current non-smokers with levels <2.77 ng/mg, and 5) self-reported current cigarette smokers with nicotine levels ≥2.77 ng/mg versus self-reported current non-smokers with levels <2.77 ng/mg. RRs and corresponding 95% confidence intervals (95% CIs) were reported for each of these comparisons to estimate the effect (e.g. dual use, ENDS and cigarettes) on SGA.

## RESULTS

### Nicotine, cotinine, NNK, and NNAL concentrations

The geometric means (GMs), medians, and ranges of nicotine and cotinine among the three smoking-status groups are presented in Table 1. Nicotine GM concentrations in samples of current dual users were similar, and not lower compared to those of current smokers, 11.0 ng/mg of hair and 10.6 ng/mg of hair, respectively (p=0.58). As expected, nicotine GM concentrations in samples of smokers were significantly different from those of non-smokers (p<0.0001); similarly, GM concentrations were different for dual users and non-smokers (p=0.0007). Cotinine GM concentrations in samples of current dual users were over twice that of current smokers at 0.153 ng/mg of hair and 0.065 ng/mg of hair, respectively, although they were not statistically significantly different (p=0.40). Cotinine GM concentrations for non-smokers were statistically significantly less (undetectable) than for current dual users or current smokers. Nicotine GM concentrations were also substantially higher than cotinine concentrations in samples of current dual users and current smokers.

**Table 1:** Distribution of nicotine and other tobacco metabolites in hair among 76 pregnant women, by selfreported use of ENDS and cigarette smoking, Little Rock, Arkansas, 2015–2016

*Metabolite (units)*	*GM (95% CI)*	*Median*	*Range*	*p-value† Non-smoker referent*	p-value* Smokers referent
**Nicotine** (ng/mg)	Self-reported dual ENDS users (n=11)			0.0007	0.58
	11.0 (3.8–31.3)	9.0	0.7 – 125.6		
	Self-Reported smokers only (n=27)			<0.0001	Referent
	10.6 (6.5–17.4)	10.7	0.8 – 102.4		
	Self-reported non-users of ENDS/non-smokers (n=38)			Referent	
	1.1 (0.6–2.0)	0.83	0.1 – 44.6		
**Cotinine** (pg/mg)	Self-reported dual ENDS users (n=11)			0.0047	0.40
	0.153 (0.004–5.316)	0.671	0.019 – 20.955		
	Self-Reported smokers only (n=27)			<0.0001	Referent
	0.065 (0.009–0.465)	0.610	0.037 – 6.106		
	Self-reported non-users of ENDS/non-smokers (n=38)			Referent	
	0.000 (0.000–0.001)	0.000	0.001 – 1.713		
**NNK** (pg/mg)	Self-reported dual ENDS users (n=11)			0.03^a^	0.18^a^
	0.213 (0.006–7.672)	6.095	0.000 – 105.163		
	Self-Reported smokers only (n=27)			0.002^a^	Referent
	0.131 (0.019–0.888)	1.299	0.000 – 27.192		
	Self-reported non-users of ENDS/non-smokers (n=38)			Referent	
	0.003 (0.001–0.011)	0.000	0.000 – 42.276		
**NNAL** (pg/mg)	Self-reported dual ENDS users (n=11)			0.22^a^	0.20^a^
	0.030 (0.002–0.395)	0.135	0.000 – 1.863		
	Self-reported smokers only (n=27)			0.74^a^	Referent
	0.005 (0.001–0.025)	0.000	0.000 – 1.081		
	Self-reported non-users of ENDS/non-smokers (n=38)			Referent	
	0.004 (0.001–0.013)	0.000	0.000 – 0.929		

GM: geometric mean.

† Non-concurrent smokers is the referent group.

* Concurrent smokers is the referent group.

a Wilcoxon rank sum.

NNK and NNAL were not consistently detected in the hair samples, so medians were calculated instead of GMs; NNK and NNAL exposures are summarized with medians and ranges (Table 1). NNK was detected in hair samples of 78% of dual users, 56% of cigarette smokers, and 20% of non-smokers. The median NNK level in hair samples of current dual users was approximately 5 times greater than that of current smokers; however, this difference was not statistically significant (Wilcoxon rank-sum p=0.18). NNAL was detected in hair samples of 67% of current dual users, 49% of current smokers, and 50% of non-smokers (χ^2^=0.9751, DF=2; p=0.61). The median NNAL level in hair samples was also greater for current dual users compared to current smokers, although not statistically significantly different (Wilcoxon rank-sum p=0.50).

### Smallness for gestational age (SGA)

The risk of SGA among self-reported current dual ENDS users was 27% compared to 8% among self-reported current non-smokers. The crude RR for self-reported current dual ENDS compared to self-reported non-smokers was 3.5 (95% CI: 0.8–14.8; p=0.11), and the RR adjusted for maternal age was 3.9 (95% CI: 0.9–16.2; p=0.06). The risk of SGA among self-reported smokers was 26%; the corresponding crude and adjusted RR for SGA for self-reported current smokers compared with self-reported non-smokers were 3.3 (95% CI: 0.9–11.6; p=0.08) and 3.9 (95% CI: 1.1–13.6; p=0.03), respectively. The offspring of women who had hair samples with nicotine levels greater than the cutoff (2.77 ng/mg) had a statistically significant crude and adjusted 7.8 and 7.7 times risk of giving birth to an SGA neonate relative to those with nicotine levels below the cutoff (Table 2). When we examined the crude and maternal age adjusted RRs using self-reported current smoking and current ENDS dual use consistent with the hair nicotine levels, we observed that current ENDS dual users had greater than 8 times the risk of giving birth to a neonate with SGA compared to non-smokers, which was similar to the large RR observed for current smokers. The addition of education, race/ethnicity to the log-binomial models did not alter the estimated RRs and so they were not included in the final models.

**Table 2:** Risk ratios for smallness for gestational age among 76 pregnant women based on self-reported ENDS/smoking status and hair nicotine, Little Rock, Arkansas, USA, 2015–2016

*ENDS/smoking status*	*n*	*SGA† n*	*Risk %*	*Risk ratio*	*95% CI*	*p*
**By self-report**						
Non-users/non-smokers	38	3	7.9	1 (referent)		
Current ENDS dual users	11	3	27.3			
Crude				3.5	0.8–14.8	0.11
Adjusted*				3.9	0.9–16.2	0.06
Current smokers	27	7	25.9			
Crude				3.3	0.9–11.6	0.08
Adjusted*				3.9	1.1–13.6	0.03
**By hair nicotine level**						
<2.77 ng/mg	30	1	3.3	1 (referent)		
≥2.77 ng/mg	46	12	26.1			
Crude				7.8	1.1–57.1	0.01
Adjusted				7.7	1.1–56.0	0.01
**By self-reports confirmed by hair nicotine levels**						
Non-users/non-smokers	25	1	4.0	1 (referent)		
Current ENDS dual users	9	3	33.3			
Crude				8.3	1.0–70.2	0.05
Adjusted				8.3	1.0–69.1	0.05
Current smokers	24	7	29.2			
Crude				7.3	1.0–54.9	0.05
Adjusted				7.8	1.0–59.0	0.05

† Adjusted for gestational age and fetal sex.

* Adjusted for maternal age.

## DISCUSSION

The introduction and marketing of ENDS has changed the method of delivery and behaviors involved in nicotine consumption, adding a layer of complexity to the study of tobacco use^37^. Social media marketing of ENDS often promotes these devices as ‘safer’ than regular cigarettes, and often claims are made that ENDS ‘aid’ smoking cessation^38^. A higher percentage of current smokers than non-smokers in Arkansas believe they can reduce the harmful effects of smoking by switching or substituting cigarettes with ENDS^38^.

To better understand the levels of nicotine and TSNAs in pregnant women, we measured nicotine, cotinine, NNK, and NNAL, in hair samples. We chose to measure the biomarker concentrations in hair because they represent cumulative exposure of 3 months or more. Use of a cumulative exposure assessment was meant to more accurately identify women who, while pregnant, were exposed to tobacco smoke or ENDS aerosols during early fetal development. This is of interest because smoking behavior typically changes before a woman becomes pregnant or before she is aware of the pregnancy^39^. In addition, metabolism changes drastically during pregnancy, which makes it difficult to interpret serum and urine levels of nicotine metabolites, which have relatively short half-lives. Measuring cumulative exposures in hair samples overcomes these difficulties and provides relatively accurate measurements of cumulative internal doses for the 3 months prior to sampling (for our study, this typically included the final portion of the first trimester and the early days of the second trimester).

The GM for nicotine in hair samples of pregnant smokers are in agreement with reports of studies that examined nicotine in the hair of non-pregnant smokers^38^. Surprisingly, instead of observing lower levels of nicotine in dual ENDS users in our population of pregnant women, we found that nicotine, cotinine, NNK, and NNAL levels, in hair samples of dual users were consistently higher (although not statistically significantly higher) than those in hair samples of smokers. This was unexpected because, if smokers use cigarettes to satisfy their nicotine cravings, and they are substituting ENDS for cigarettes, then it is expected that they will use both products to reach the same level of nicotine intake^39-41^. Nicotine intake among ENDS users may be enhanced by sublingual absorption, compared to inhaled smoke from cigarettes, which is mostly absorbed through the lungs. Our results suggest that pregnant women using ENDS while continuing to smoke cigarettes may expose their babies to similar or greater amounts of nicotine and other toxicants than women who only smoke cigarettes.

The incidence of smallness for gestational age (SGA) was similar for pregnant self-reported dual users (27%) and pregnant self-reported smokers (26%). The estimated adjusted RRs for SGA were also similar for pregnant dual users and pregnant smokers relative to non-smokers. This similarity in RRs remained after restricting the classification of self-reported dual users and smokers to those confirmed by hair nicotine levels ≥2.77 ng/mg and self-reported non-smokers confirmed by nicotine levels <2.77 ng/mg. The estimated risk of SGA among confirmed dual users was over 8 times that for confirmed non-smokers.

### Limitations and strengths

There are several limitations to our study. First, our sample was small. Future studies with more participants should be able to determine whether the NNK-to-nicotine ratio of ENDS users is different from that of cigarette users. Second, we were unable to collect e-liquids used by our participants, which would have allowed us to assess their nicotine and NNK content and to determine whether they correlated with levels detected in hair samples. Third, only one participant reported sole use of ENDS and was excluded from the analysis. All remaining ENDS users also smoked cigarettes. Therefore, we were not able to estimate the average exposure to nicotine, cotinine, or TSNAs for ENDS-only users.

Our study has several strengths. First, our study used a prospective cohort design. Second, our study filled a gap in the literature by measuring nicotine, cotinine, and TSNAs in hair, hence with longer half-lives, instead of relying solely on self-reports in a study population of pregnant women, including women who reported using ENDS. Third, we estimated the effect of nicotine, cotinine, and TSNAs measured during the end of the first trimester or early in the second trimester on the birth SGA outcome. One previous study used nicotine levels in hair to assess the association between tobacco exposure and the risk of SGA as a birth outcome. Unfortunately, that study used hair specimens collected during delivery, and thus assessed only the effects of exposure during the last trimester^42^.

## CONCLUSIONS

The use of nicotine products other than cigarettes has recently increased over time. The current study provides novel data on hair nicotine levels for pregnant women who were exposed to nicotine using ENDS as well as cigarettes, and provides estimates for the association between tobacco exposure during pregnancy and smallness for gestational age. We found that exposure to nicotine, cotinine, NNK, or NNAL, was not lower for pregnant women who were dual users (i.e. ENDS and cigarettes) compared with those who were cigarette smokers. Dual users were exposed to NNK, a known human carcinogen, at the same or greater levels as smokers were, which raises concerns about the long-term adverse effects of ENDS. Furthermore, the risk of smallness for gestational age for offspring of pregnant dual users was similar to that for offspring of pregnant smokers. Future studies, preferably with larger numbers of ENDS-only users, are needed which examine the effect of nicotine product use during pregnancy on birth outcomes.

## CONFLICTS OF INTEREST

The authors declare that they have no competing interests, financial or otherwise, related to the current work. U. Ekanem reports grants from Arkansas Department of Health, personal fees from University of Arkansas for Medical Sciences Fay W. Boozman College of Public Health, grants from National Institutes of Health (NIH) - National Center for Research Resources and National Center for Advancing Translational Sciences, during the conduct of the study. The rest of the authors have also completed and submitted an ICMJE form for disclosure of potential conflicts of interest.
